# Generalized tendency to make extreme trait judgements from faces

**DOI:** 10.1098/rsos.220172

**Published:** 2022-11-23

**Authors:** Atsunobu Suzuki, Saori Tsukamoto, Yusuke Takahashi

**Affiliations:** ^1^ Department of Psychology, Graduate School of Humanities and Sociology, The University of Tokyo, 7-3-1 Hongo, Bunkyo-ku, Tokyo 113-0033, Japan; ^2^ Department of Psychology, Aichi Gakuin University, 12 Araike, Iwasaki-cho, Nisshin, Aichi 470-0195, Japan; ^3^ Division of Cognitive Psychology in Education, Graduate School of Education, Kyoto University, Yoshida-honmachi, Sakyo-ku, Kyoto-shi, Kyoto 606-8501, Japan

**Keywords:** face, trait inference, individual differences, physiognomic belief, facial emotion recognition, stereotypes

## Abstract

People differ in their tendency to infer others' personalities and abilities from their faces. An extreme form of such face-based trait inference (FBTI) is problematic because of its unwarranted impact on real-world decision making. Evolutionary perspectives on FBTI suggest that its inter-individual variation would be *trait-specific*: e.g. those who make extreme face-based inferences about trustworthiness may not necessarily do so about dominance. However, there are several psychological variables that can increase the FBTI extremity across traits. Here, we show that there is a generalized individual tendency to make extreme FBTI across traits, in support of the latter view. We found that the degrees of extremity of face-based inferences about seven traits had high cross-trait correlations, constituting a general factor. This *generalized FBTI extremity* had good test–retest reliability and was neither an artefact of extreme nor socially desirable response biases. Moreover, it was positively associated with facial emotion recognition ability and tendencies to believe physiognomy and endorse stereotypes. Our results demonstrate that there are individuals who have a temporally stable disposition to draw extreme conclusions about various traits of others from facial appearance as well as their psychological characteristics.

## 1. Introduction

There is growing attention being paid to individual differences in how people make trait inferences from faces [1]. Until recently, the literature had focused on a high degree of agreement among people on the first impressions of personality and ability derived from faces [2]. This emphasis on high agreement in face-based trait inference (FBTI) is now being reconsidered [3–5]. For instance, variances in face-derived impressions of trustworthiness, dominance and youthful/attractiveness were explained more by perceiver differences (approx. 23%) than by face differences (approx. 15%), implying that some people consistently made extreme judgements across faces on the respective impression dimensions [5].

An extreme form of FBTI is problematic because of its unwarranted impact on real-world decision making. FBTI is known to affect consequential real-world decisions, such as voting and criminal sentencing [2,6,7], while its accuracy, if any, is, at most, only modest and lacking practical utility [6,8–11]. Additionally, extreme first impressions based on little information (e.g. face photos) are often accompanied by high confidence [12], and confident beliefs tend to have low malleability [13] and affect others’ evaluations [14]. Extreme FBTI is therefore troublesome and merits a deeper understanding.

Do those who make extreme face-based judgements on a certain trait tend to make extreme judgements on other traits as well? This question is important because such individuals should be a prime target for intervention to reduce the biasing impact of facial appearance on interpersonal judgements and choices [6,15,16], but this has yet to be answered. The present study thus examines if there is an overall individual tendency to make extreme face-based judgements across traits, which is termed *the generalized FBTI extremity*. The FBTI extremity is the strength of the differentiation/polarization of trait judgements based on faces. For instance, there are stereotypically trustworthy- and untrustworthy-looking faces for which people tend to assign higher and lower trustworthiness ratings, respectively [17]. The extent to which ratings diverge from stereotypically trustworthy- to untrustworthy-looking faces has been used to assess social cognition in neurological [18], ageing [19] and developmental research [20]. In accordance with previous work, we focus on a difference score computed by subtracting the mean of the trustworthiness ratings of stereotypically untrustworthy-looking faces from that of stereotypically trustworthy-looking faces for each participant. The higher the score, the greater was the FBTI extremity. Stereotypical faces also exist for traits other than trustworthiness [2]; hence, the FBTI extremity can be defined in the same fashion across traits.

It is to be noted that the FBTI extremity is conceptually different from ‘agreement scores’ of facial impressions. For instance, Sutherland *et al*. [21] had participants rate 100 faces on trustworthiness, dominance and attractiveness impressions, and computed the agreement scores by correlating each participant's ratings with the population averages. The higher the correlation, the greater the extent to which facial impressions of the participant matched the face stereotypes commonly held in the population. An intuitive example that distinguishes an extremity from an agreement is as follows. Suppose that the population means of the trustworthiness rating for a stereotypically trustworthy-looking face and a stereotypically untrustworthy-looking face are *M*_T_ and *M*_U_, respectively. One participant shows a difference in the trustworthiness rating between the two faces that is somewhat larger than *M*_T_ – *M*_U_, while another participant shows the difference somewhat smaller than *M*_T_ – *M*_U_. In this situation, the two participants would be assigned similar agreement scores (i.e. the degrees to which their facial impressions are consistent with face stereotypes would be deemed as equally moderate), whereas the former participant would be scored higher in FBTI extremity than the latter participant. In short, the FBTI extremity is akin to a measure of the steepness of the change in one's facial impressions with facial stereotypes.

The idea of generalized FBTI extremity seems incompatible with the evolutionary accounts of FBTI, which assume specialized functions and mechanisms for judgements of specific traits [22–24]. For example, judgements of trustworthiness and dominance—two major traits inferred from faces [17,25,26]—are proposed to deal with the unique adaptive problems of choosing the right leaders in peacetime and wartime, respectively [24]. Under such distinct selection pressures, different neural circuits may have evolved to assess the respective traits [27,28]. If individual differences in judging specific traits reflect variations in specialized neurocognitive mechanisms, they should have little correlation with each other.

In contrast with the above view, here we propose that the FBTI extremity would show high cross-trait correlations and that a generalized FBTI extremity would exist. Our proposal is based on the consideration that several psychological variables can interrelate FBTI extremity across traits, which are detailed below.

First, lay beliefs in favour of FBTI may underlie generalized FBTI extremity. A cross-cultural study in the United States (USA) and Japan showed that those who believed that faces reveal a certain trait tended to extend their belief to other traits [29]. This comprehensive belief that various traits can be inferred from faces is termed *physiognomic belief*. The same study also demonstrated that people with a strong physiognomic belief are prone to making extreme face-based judgements of trustworthiness, dominance and competence. That is, the physiognomic belief can foster the FBTI extremity across traits, thereby possibly contributing to the emergence of a generalized FBTI extremity.

Second, individual differences in recognizing facial expressions may have a significant influence on trait judgements based on faces. Studies examining trait inferences from neutral faces have shown that the structural resemblances of neutral faces to certain facial expressions are a major determinant of the attribution of diverse traits [17,30,31]. The overgeneralization hypothesis of FBTI [22] explains these results as being due to human sensitivity with regard to detecting subtle facial expressions [32] and to the human tendency to regard momentary mental states as a manifestation of enduring characters [33]. That is, people may perceive someone as being trustworthy if they discern a slight smile in that person's face [17,34] and interpret this transient affection as evidence of stable benevolence. This sort of trustworthiness attribution should be enhanced by increasing sensitivity to happy facial expressions. Indeed, people are far from equally sensitive to facial expressions; rather, there is considerable variability in their recognition [35,36]. A possibility is that those with greater ability to notice subtle expressive differences among faces tend to provide more differentiated trait ratings [20], thus scoring high on the FBTI extremity.

Third, people who generally endorse stereotypes—beliefs about the traits commonly held by members of specific social categories—are expected to make extreme face-based judgements regarding a range of traits, since stereotype application, or category-based trait inference, should provide a broad basis for FBTI. For instance, happy faces (smiles), baby-facedness and facial femininity, respectively, lead to consensual judgements of high trustworthiness, low dominance and low competence, which may reflect the shared beliefs that smiling people are good-tempered [17,33], babies are naive and weak [37], and women are less competent than men [38]. It follows that if certain people have a greater inclination to endorse stereotypes across social categories, they would be more likely to make extreme face-based inferences across traits. Previous studies have indeed demonstrated individual differences in a general tendency to acknowledge and use stereotypes in daily life, the so-called *acceptance of stereotyping* [39], which exacerbates negative attitudes toward others belonging to an array of social categories [40].

Finally, *cognitive misers* who rely on and do not challenge their intuition [41] may tend to make extreme face-based judgements across traits. FBTI involves little time and effort [2] and accompanies affective gut feelings [19], leading to subjective experiences of ‘intuiting’ [42]. Moreover, people are known to differ in their faith in intuitive responses [43] and vary in their (un)willingness and (in)ability to detect and correct erroneous hunches [41,43,44]. High cognitive miserliness would thus be accompanied by uncritical acceptance of the prototypical facial impressions that come to mind first, consequently enhancing the FBTI extremity.

In summary, several different psychological factors converge to predict the existence of a generalized FBTI extremity, while empirical investigation of this possibility is scarce. Therefore, we performed two studies (Studies 1 and 2) to test the hypotheses that there would be a generalized tendency to make extreme face-based inferences across traits and that this generalized FBTI extremity would be positively related to physiognomic belief, facial emotion recognition ability, stereotype endorsement and cognitive miserliness. The hypotheses and analysis plans were preregistered on the Open Science Framework, which can be accessed via the following link, along with the study materials, datasets and analysis codes: https://osf.io/axtvz.

## 2. Study 1

### 2.1. Objectives

Study 1 was designed to provide evidence for a generalized FBTI extremity and its relationship with physiognomic belief. Participants rated their impressions of a number of faces on several trait dimensions and completed a questionnaire on the physiognomic belief. The temporal stability of the generalized FBTI extremity was also examined by asking some of the participants to complete the same survey twice.

### 2.2. Methods

#### 2.2.1. Participants

Three hundred native English-speaking US residents aged 20–49 years old were recruited for an online survey via Prolific Academic (https://www.prolific.co). The sample size was predetermined to detect *r* = 0.2 with 90% power (*α* = 0.05), assuming an effective response rate of 90%. Fourteen responses were excluded based on the preregistered criteria. The remaining data (156 men, 130 women; *M* ± s.d. of age = 31.74 ± 7.91; 66% White, 8% Black, 8% Asian, 4% Hispanic, 14% other ethnicities/multi-ethnic) were considered effective and analysed.

The respondents were invited to take a follow-up survey about two months later; the survey was stopped after 120 responses were collected. This sample size enabled us to estimate a correlation with a precision of ± 0.1 in terms of the half-width of a 95% confidence interval (CI) [45], assuming an effective response rate of 90% and a true correlation of 0.7. Twelve participants who met the same exclusion criteria as in the main survey or whose answers to the demographic questions were incongruent between the two surveys were discarded. The remaining data (64 men, 44 women; *M* ± s.d. of age = 32.23 ± 8.03; 66% White, 10% Asian, 10% Black, 5% Hispanic, 9% other ethnicities/multi-ethnic) were used for test–retest reliability analysis. This follow-up survey was not preregistered.

#### 2.2.2. Procedure and tasks

##### 2.2.2.1. Overview

The procedure and tasks were the same in the main and follow-up surveys. Participants accessed the survey website constructed by Qualtrics software (https://www.qualtrics.com). After giving informed consent, they first answered basic socio-demographic questions and then undertook the main tasks assessing FBTI extremity and the physiognomic belief, the order of which was randomized across participants. The item (stimulus) order in each task was also randomized unless otherwise noted.

##### 2.2.2.2. Face-based trait inference extremity

**Figure 1.**
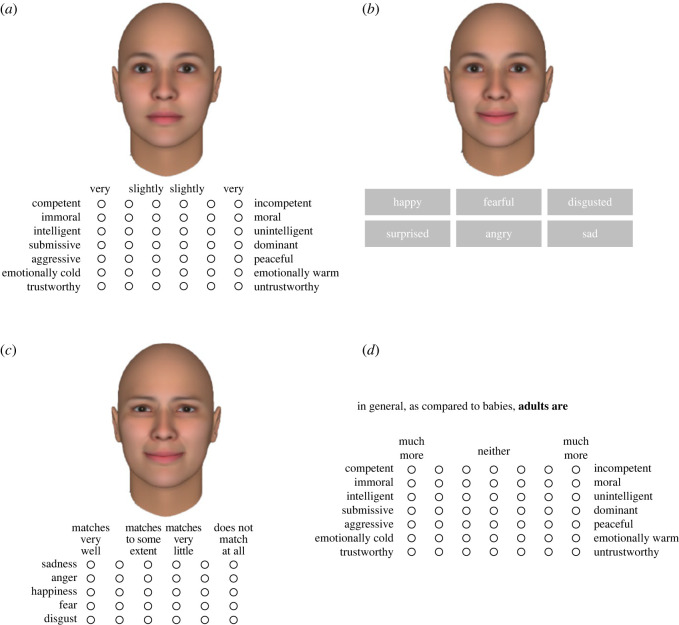
Illustration of the rating/identification tasks used in Studies 1 and 2: (*a*) face-based trait-rating task; (*b*) facial emotion identification task; (*c*) facial emotion-rating task and (*d*) category-based trait-rating task. Sample faces in this figure are not of real people; they are computer graphics images created using FaceGen Modeller (https://facegen.com). Photos of real people's faces [46–48] were used as stimuli in the actual surveys, which are not presented here for copyright and privacy reasons.

A face-based trait-rating task was used to measure the construct. In each trial, participants rated their impressions about a person whose face was presented on the screen using seven 6-point semantic differential (SD) scales, ranging from *very aggressive* to *very peaceful*, *very competent* to *very incompetent*, *very submissive* to *very dominant*, *very intelligent* to *very unintelligent, very immoral* to *very moral*, *very trustworthy* to *very untrustworthy* and *very emotionally cold* to *very emotionally warm* (figure 1*a*). These traits have been of great interest in FBTI research [2] and/or are considered as fundamental person perception dimensions [49]. The scale anchors were fixed as described above, so that desirable traits appeared almost evenly on the left and right sides, while the order of the scales was randomized across trials. Neutral faces of 60 Caucasian individuals (equally split between men and women) from the Karolinska Directed Emotional Faces database [46] were presented.

The extremity of FBTI was scored for each trait. Taking trustworthiness as an example, the ratings (1 = *very untrustworthy* to 6 = *very trustworthy*) were averaged for each face, and the top and bottom 10 faces that received the highest and lowest mean ratings, respectively, were selected separately from among male and female faces. Then, for each participant, the ratings were averaged across the top- and bottom-ranked 20 faces. The extremity score of the face-based trustworthiness inference was then computed by subtracting the latter average from the former one. Extremity scores for other trait judgements were defined in the same way, and the averages of the trait-specific scores were treated as an overall index of FBTI extremity. Our preregistration included another method to score FBTI extremity, whereby the ratings of 6, 5, 4, 3, 2 and 1 were recoded into 3, 2, 1, 1, 2 and 3, respectively. Analysis of this alternative score is omitted here for simplicity but is included in electronic supplementary material, text S1.

##### 2.2.2.3. Physiognomic belief

The Physiognomic Belief Scale [29] was used to measure this construct. It included 14 target items, each stating that a specific trait can be inferred from the face (e.g. ‘I know a trustworthy person when I see their face’). Participants rated how closely each item matched their own thoughts on a scale ranging from 1 (*disagree*) to 4 (*agree*). The mean of the ratings was taken as a measure of the strength of each participant's physiognomic belief. The questionnaire also contained four control items that should normally be endorsed (e.g. ‘I can tell babies and the elderly apart by their faces’) or rejected (e.g. ‘I can guess a person's address correctly when I see their face’); these were used to screen out unusual (i.e. possibly inattentive) responses.

### 2.3. Results and discussion

Below, we report partial correlations in which participants' age and sex (coded as male = 1 and female = 2) were controlled, consistent with the analytic approach adopted in Study 2 and relevant past work [29]. While this deviates from our preregistration to examine zero-order correlations, the preregistered analysis yielded similar results to those presented here.

Partial correlations among the trait-specific extremity scores of the FBTI ranged from 0.503 to 0.825 (table 1). Both the parallel analysis and the minimum average partial (MAP) test [50] favoured a one-factor solution to account for the correlational pattern. The single factor explained 64.0% of the total variance, and the loading of each trait-specific score ranged from 0.710 to 0.883 (table 1). The results supported our hypothesis that there would be a generalized tendency toward extreme FBTI across traits. Thus, the overall index of the FBTI extremity was computed by averaging all trait-specific scores (Cronbach's *α* = 0.928). This generalized FBTI extremity had an expected but relatively small positive correlation (partial *r* = 0.131, 95% CI [0.014, 0.243]) with the Physiognomic Belief Scale score (*α* = 0.913). In addition, the generalized FBTI extremity had a high correlation between the main and follow-up surveys (partial *r* = 0.799, 95% CI [0.718, 0.859]), indicating that this individual characteristic is temporally stable.

**Table 1.** Partial correlations among the trait-specific extremity scores on the face-based trait-rating task and results of exploratory factor analysis of the scores. Partial correlations were controlled for participants' age and sex in Study 1 and for participants’ age, sex, extreme response bias and social desirability bias in Study 2. The 95% CIs for the respective partial correlations were omitted to avoid visual clutter. In brief, the minimum lower bounds of the 95% CIs were 0.411 (Study 1) and 0.404 (Study 2), and the maximum upper bounds were 0.859 (Study 1) and 0.855 (Study 2).

trait	partial correlation							factor loading	
	upper diagonal: Study 1; lower diagonal: Study 2								
	1	2	3	4	5	6	7	Study 1	Study 2
1. aggressiveness	–	0.555	0.719	0.586	0.755	0.720	0.737	0.854	0.789
2. competence	0.571	–	0.503	0.825	0.600	0.614	0.520	0.718	0.815
3. dominance	0.684	0.524	–	0.522	0.571	0.587	0.574	0.710	0.668
4. intelligence	0.552	0.821	0.521	–	0.633	0.652	0.535	0.748	0.782
5. morality	0.670	0.668	0.493	0.622	–	0.824	0.689	0.883	0.840
6. trustworthiness	0.667	0.700	0.516	0.660	0.770	–	0.685	0.878	0.863
7. warmth	0.693	0.572	0.587	0.534	0.671	0.684	–	0.789	0.781
	proportion of variance explained							0.640	0.630

Non-preregistered exploratory analysis was also conducted to determine whether our participants provided unusually undifferentiated facial impressions, since all traits were rated at once (figure 1*a*). The means of the seven trait ratings were computed for each face and subjected to principal component analysis [17]. The parallel analysis and MAP test favoured two- and three-component solutions, respectively, and the former solution was chosen for parsimony and interpretability. The two components can be interpreted as representing trustworthiness and dominance, accounting for 75.1% and 22.5% of the variance, respectively (table 2). Thus, the participants' face impression space was no simpler than the well-established two-dimensional model [17,25,26].

**Table 2.** Results of the principal component analysis of the seven trait ratings for the 60 faces. Factor loadings greater than 0.30 are in italics. In Studies 1 and 2, the first and second components can be interpreted as representing the trustworthiness and dominance dimensions, respectively, in the two-dimensional model of facial impressions [17].

trait	loading					
	Study 1			Study 2		
	1	2	3	1	2	3
aggressiveness	*−0.888*	*0.435*	0.074	*−0.917*	*0.364*	0.104
competence	*0.809*	*0.577*	−0.075	*0.767*	*0.628*	−0.099
dominance	*−0.544*	*0.811*	0.202	*−0.609*	*0.751*	0.243
intelligence	*0.794*	*0.572*	−0.188	*0.761*	*0.623*	−0.155
morality	*0.986*	−0.003	0.084	*0.985*	< 0.001	0.104
trustworthiness	*0.986*	0.096	0.064	*0.986*	0.075	0.096
warmth	*0.938*	−0.198	0.255	*0.919*	−0.261	0.262
proportion of variance explained	0.751	0.225	0.024	0.747	0.225	0.028

## 3. Study 2

### 3.1. Objectives

Study 2 had two main objectives. The first was to provide further evidence of generalized FBTI extremity by ruling out the possibility that it is an artefact of response biases. Study 1 demonstrated high cross-trait correlations for the extremity of face-based inferences. However, these correlations may be spurious and may have been caused by response biases. That is, people who habitually choose extreme categories on the rating scale independent of item content [51] tend toward extreme FBTI across traits. By contrast, those inclined to give socially desirable responses [52] may approach FBTI modestly, in keeping with the popular adage ‘Don't judge a book by its cover'. Study 2 aimed to demonstrate a generalized FBTI extremity considering these confounders. The second objective was to examine the hypothesis that the generalized FBTI extremity was positively related to facial emotion recognition ability, stereotype endorsement and cognitive miserliness, as well as to replicate its link with physiognomic belief. To accomplish these goals, we had participants complete a battery of tasks to assess each construct.

### 3.2. Methods

#### 3.2.1. Participants

Study 2 consisted of two successive surveys: Surveys I and II. Before data collection, we planned to gather at least 325 responses to both surveys. The sample size enables us to detect *r* = 0.2 with 90% power (*α* = 0.05), assuming an effective response rate of 80%. Native English speakers residing in the USA and aged 20 to 49 years old were recruited via Prolific Academic. Four hundred people completed Survey I, and of these, 336 participated in Survey II. Among those who completed both surveys, 24 were excluded based on the preregistered criteria and due to incongruent answers to the demographic questions in the two surveys. The final sample thus included 312 respondents (147 men, 165 women; *M* ± s.d. of age at Survey I = 31.12 ± 7.70; 73% White, 9% Asian, 7% Black, 2% Hispanic, 9% other ethnicities/multi-ethnic).

#### 3.2.2. Procedure and tasks

##### 3.2.2.1. Overview

Participants accessed the survey website constructed by Qualtrics software. The survey consisted of two parts, and participants completed them at an interval of one–three weeks. Each survey started with obtaining informed consent and basic information from participants, similar to Study 1. Survey I then measured FBTI extremity, physiognomic belief and response biases. In Survey II, facial emotion recognition ability, stereotype endorsement and cognitive miserliness were measured. The order of the tasks in each survey was randomized across participants. The item (stimulus) order in each task was also randomized unless otherwise noted.

##### 3.2.2.2. Face-based trait inference extremity

FBTI extremity was measured using the same task as in Study 1, except that two attention-check items were given upon completion of one-third and two-thirds of the task. The attention-check items required that participants choose a specific number on a scale ranging from 1 to 6 (e.g. ‘Please choose ‘1’ for this question’).

##### 3.2.2.3. Physiognomic belief

The strength of the physiognomic belief was measured using the Physiognomic Belief Scale [29], as in Study 1, and using a questionnaire developed by Jaeger *et al.* [53]. Jaeger's questionnaire of physiognomic belief asked participants to imagine seeing a stranger's passport photo and indicate how much they agreed with three items, each referring to the subjective (in)validity of FBTI (e.g. ‘I do not believe that the person's personality is reflected in their face’), on a scale ranging from 1 (*strongly disagree*) to 7 (*strongly agree*). Thus, a major difference between the two questionnaires is that the items of the Physiognomic Belief Scale mention specific traits whereas those of Jaeger's questionnaire of physiognomic belief do not. The mean of each questionnaire's ratings was used as a separate index of physiognomic belief.

##### 3.2.2.4. Extreme response bias

The Extreme Response Style Measure [51] was used to measure the construct. Participants indicated how much they agreed with 16 unrelated statements (e.g. ‘I am a homebody,’ ‘I eat more than I should’) on a scale ranging from 1 (*strongly disagree*) to 6 (*strongly agree*). Extreme response bias was computed as the proportion of statements for which an endpoint value on the scale (i.e. 1 or 6) was chosen.

##### 3.2.2.5. Social desirability bias

The Impression Management subscale from the short form of the Balanced Inventory of Desirable Responding [52,54] was used to measure this construct. It included eight items describing either socially desirable (e.g. ‘I never cover up my mistakes’) or undesirable behaviours (e.g. ‘I have said something bad about a friend behind his or her back’). Participants indicated to what extent each item was personally applicable on a scale ranging from 1 (*not true*) to 7 (*true*). The number of answers for which desirable and undesirable behaviours were rated as highly applicable (i.e. 6 or 7 was chosen) and highly inapplicable (1 or 2), respectively, was defined as a measure of social desirability bias.

##### 3.2.2.6. Facial emotion recognition ability

The construct was measured using two tasks. One task was a facial emotion identification task. In each trial, participants viewed a photo of a facial expression and identified the expressed emotion from a list of six emotion words: happy, surprised, fearful, angry, disgusted and sad (figure 1*b*). The emotion words were displayed in this fixed order. A total of 48 photos from the Japanese and Caucasian Facial Expressions of Emotion database [47] were presented, consisting of prototypical facial expressions of the six emotions presented by eight individuals (an equal split between male and female and Caucasian and Japanese). An attention-check question was posed halfway through the task; digits ranging from 1 to 6 were displayed and participants were asked to choose 1. Performance on the facial emotion identification task was first scored for each emotion as Cohen's *κ*, which is a correct response rate corrected for response bias [55]. The average of the emotion-specific Cohen's *κ*s was used as an overall index of facial emotion recognition ability.

The other task was a facial emotion-rating task. In each trial, participants viewed a photo of a facial expression and rated how well five emotion words (sadness, anger, happiness, fear and disgust) described the affective experiences encoded in the facial expression (figure 1*c*). Five 6-point scales, ranging from 1 (*does not match at all*) to 6 (*matches very well*), were used to rate each emotion. The scales were situated below each face, and the order of the emotions was fixed as given above. Twenty-five photos were presented, all depicting the face of a Caucasian male called JJ from the Pictures of Facial Affect [48]. Five of the images showed prototypical facial expressions of the five emotions. The remaining 20 photos were ambiguous facial expressions generated by morphing [56]. Specifically, for each of the 10 possible pairs of the five prototypical expressions, two intermediate expressions were synthesized by blending the prototypes in a 3 : 2 ratio (e.g. 60% sadness and 40% anger) and vice versa using FantaMorph (https://www.fantamorph.com). Unlike the identification task, the surprised expression was not included because mixing it with another expression occasionally caused noticeable distortion in the mouth region due to its wide-open mouth. An attention-check question was given halfway through the task; digits ranging from 1 to 6 were displayed, and participants were asked to choose 6.

Performance on the facial emotion-rating task was first scored for each emotion. Taking ‘fear’ as an example, the ratings were averaged separately across the nine fear-containing faces (i.e. one prototypical expression of fear and eight blended expressions containing it) and across the other 16 fear-non-containing faces. Then, the difference in the mean rating between the fear-containing and non-containing faces was taken as the score of fear recognition. Recognition of the other emotions was scored in the same way, and the average of the emotion-specific scores was used as a composite measure of facial emotion recognition ability.

##### 3.2.2.7. Stereotype endorsement

The construct was assessed using two tasks. One task was the Acceptance of Stereotyping Questionnaire [39]. It contained 12 items pertaining to subjective validity, usefulness and the necessity of stereotypes (e.g. ‘Stereotypes are useful in daily life even though they are not always correct’). Participants rated how closely each item matched their own thoughts on a scale ranging from 1 (*strongly disagree*) to 6 (*strongly agree*). The mean of the ratings was used as a measure of the tendency to endorse stereotypes in general.

The other task was a category-based trait-rating task, requiring participants to make comparative trait judgements between pairs of contrasting social groups. The following three pairs of categories were compared, given their close links with FBTI: people with happy versus angry faces [17,33], babies versus adults [37] and females versus males [38]. In each trial, target and reference categories were presented at the top of the screen (e.g. ‘In general, as compared to males [reference category], females [target category] are:’), and below were the seven 7-point SD scales, ranging from *much more aggressive* to *much more peaceful*, *much more competent* to *much more incompetent*, *much more submissive* to *much more dominant*, *much more intelligent* to *much more unintelligent*, *much more immoral* to *much more moral*, *much more trustworthy* to *much more untrustworthy* and *much more emotionally cold* to *much more emotionally warm* (figure 1*d*). The scale anchors were fixed as described above, so that desirable traits appeared almost evenly on the left and right sides; the order of the scales was randomized across trials, similarly to the face-based trait-rating task. In addition to the three pairs of interest, two filler pairs (rich versus middle-class people, relatives and friends versus strangers) were included. Each pair of categories was presented twice, with the opposite allocation of target and reference categories, resulting in a total of 10 trials.

**Figure 2.**
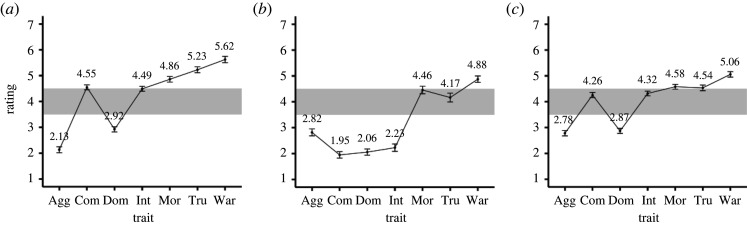
Means and 95% CIs of the comparative trait ratings between pairs of contrasting social categories. Ratings (the values on the ordinate) indicate the extent to which the people in the target category (people with happy faces, babies or females) were judged to possess each trait compared with the reference category (people with angry faces, adults or males). Stereotypes that certain traits are more/less applicable to a target category of people were defined to exist if the mean ratings of those traits were greater/lower than the scale's midpoint of 4 by 0.5 (i.e. if the mean ratings fell outside the shaded area in the figure). (*a*) Happy > angry, (*b*) babies > adults and (*c*) females > males. Agg = Aggressiveness; Com = Competence; Dom = Dominance; Int = Intelligence; Mor = Morality; Tru = Trustworthiness; War = Warmth.

The strength of stereotype endorsement was quantified as follows. First, the ratings were recoded, so that people with happy faces, babies or females constituted the target category; that is, the ratings were reversed when people with angry faces, adults or males were allocated to the target category. Next, the ratings were averaged separately for each trait and category (figure 2). A stereotype attributing a certain trait to a certain category of people was considered existent if the mean rating of that trait for that group differed by more than 0.5 from the scale's midpoint of 4; this rule was preregistered. The ratings related to the stereotypes that were defined in this way were then extracted and recoded again, so that the values increased in a stereotype-consistent direction. For instance, because the mean rating for ‘Females are more aggressive than males' was below 3.5 (figure 2), we subtracted each participant's rating from eight, so that the recoded ratings represented the strength of agreement on the stereotype that ‘Males are more aggressive than females'. Finally, the recoded ratings of all identified stereotypes were averaged for each participant to create a composite score, which reflected the degree of endorsement of various stereotypical category–trait associations.

##### 3.2.2.8. Cognitive miserliness

This construct was measured using two tasks (three indices). One task was the Cognitive Reflection Test [44]. It consisted of three arithmetic questions (e.g. ‘A bat and a ball cost $1.10. The bat costs $1.00 more than the ball. How much does the ball cost?’), for which people readily came up with incorrect answers (i.e. 10 cents); a moment's reflection to override the intuitive answers was necessary to reach the correct answers (i.e. 5 cents). The total number of correct answers given by each participant was used as an index of cognitive reflection, that is, a reverse measure of cognitive miserliness. As this test is rather well known to the general public, participants were also asked if they had taken it before.

The other task was a 10-item version of the Rational-Experiential Inventory [43], which included two subscales. The first subscale, called Need for Cognition, consisted of five items, each describing one's preference for effortful thinking (e.g. ‘I prefer complex to simple problems’). The second subscale, called Faith in Intuition, also consisted of five items, each describing one's confidence in initial feelings and thoughts (e.g. ‘I believe in trusting my hunches’). Participants rated how much they agreed with each item on a scale ranging from 1 (*completely false*) to 5 (*completely true*). The mean ratings for the Need for Cognition and Faith in Intuition subscales were deemed as reverse and forward indices of cognitive miserliness, respectively.

### 3.3. Results and discussion

#### 3.3.1. General notes on data analysis

Partial correlations reported below were controlled for participants' age, sex (coded as male = 1 and female = 2), extreme response bias (Cronbach's *α* = 0.762) and social desirability bias (*α* = 0.695) unless otherwise specified. Analysis based on zero-order correlations yielded results similar to those presented here.

#### 3.3.2. Generalized face-based trait inference extremity

Partial correlations among the seven trait-specific extremity scores of the FBTI ranged from 0.493 to 0.821 (table 1). Both the parallel analysis and MAP test favoured a one-factor solution to account for the correlational pattern. The single factor explained 63.0% of the total variance, and the loadings of each trait-specific score ranged from 0.668 to 0.863 (table 1). Thus, the generalized tendency toward extreme FBTI across traits was observed even when extreme and socially desirable response biases were controlled for, validating the computation of an overall index by averaging the trait-specific scores (*α* = 0.932). As in Study 1, when the means of the seven trait ratings were computed for each face and submitted for principal component analysis, two components that were interpretable as representing trustworthiness and dominance were obtained (table 2).

#### 3.3.3. Physiognomic belief

Reliability coefficients for the Physiognomic Belief Scale and Jaeger's questionnaire of physiognomic belief were *α* = 0.918 and 0.817, respectively. They had a positive partial correlation of 0.649 (95% CI [0.579, 0.709]), confirming that they reflected a similar construct.

#### 3.3.4. Facial emotion recognition ability

The mean Cohen's *κ*s for the facial emotion identification task were 0.987, 0.840, 0.739, 0.829, 0.777 and 0.940 for happiness, surprise, fear, anger, disgust and sadness, respectively, indicating that participants were highly accurate in this task. The internal consistency of the six *κ* scores was *α* = 0.775. As for the facial emotion-rating task, the means of the difference scores were positive for all emotions (*M* = 2.00, 2.36, 1.69, 1.21 and 2.15 for happiness, fear, anger, disgust and sadness, respectively), indicating that participants were, on average, able to discern subtle cues embedded in morphed facial expressions. The internal consistency of the five difference scores was *α* = 0.662. Average scores across emotions computed separately for the two tasks showed a positive partial correlation of *r* = 0.357 (95% CI [0.255, 0.451]), indicating that they measured an overlapping construct.

#### 3.3.5. Stereotype endorsement

The reliability coefficient for the Acceptance of Stereotyping Questionnaire was *α* = 0.841. The category-based trait-rating task data indicated that the following stereotypes were common among the participants: people with happy faces are less aggressive, more competent, less dominant, more moral, more trustworthy and warmer than people with angry faces; babies are less aggressive, less competent, less dominant, less intelligent and warmer than adults; females are less aggressive, less dominant, more moral, more trustworthy and warmer than males (figure 2). The internal consistency of the strengths of agreement with these 16 stereotypes was *α* = 0.843. Although the scores of this questionnaire and this task were assumed to reflect a similar construct (i.e. stereotype endorsement), their correlation was around zero (*r* = 0.048, 95% CI [–0.064, 0.159]).

#### 3.3.6. Cognitive miserliness

The number of correct responses on the Cognitive Reflection Test was, on average, 1.40 (s.d. = 1.19). However, the data may need to be interpreted with caution, as 43.3% of the participants reported that they had taken the test before, and those participants did, in fact, attain higher scores (1.70 ± 1.19) than those who had not (1.18 ± 1.13; *t*_310_ = 3.937, *p* < 0.001). Reliability coefficients for the Need for Cognition and Faith in Intuition subscales from the Rational-Experiential Inventory were *α* = 0.820 and 0.882, respectively. Correlations among the three scores were relatively small (table 3), consistent with the claims that they measure distinct aspects of cognitive miserliness [41,43].

**Table 3.** Partial correlations and correlations among the measures in Study 2 and multiple regression results for the generalized FBTI extremity. Partial correlations were controlled for participants' age, sex, extreme response bias and social desirability bias. Statistically significant (partial) correlations and standardized partial regression coefficients (*β*) (*p* < 0.05) are in italics. The *R*^2^ value of the regression was 0.385.

measure	correlation and partial correlation											regression results	
	1	2	3	4	5	6	7	8	9	10		*β*	*p*
	partial correlation (upper diagonal: point estimate; lower diagonal: 95% CI)												
1. generalized FBTI extremity	–	*0.171*	0.104	*0.198*	*0.348*	0.092	*0.430*	0.106	−0.016	0.024	1	–	–
2. physiognomic belief (1)^a^	[0.061, 0.278]	–	*0.649*	*−0.155*	−0.070	*0.241*	*0.211*	−0.028	0.089	*0.363*	2	*0.148*	0.023
3. physiognomic belief (2)^b^	[−0.008, 0.213]	[0.579, 0.709]	–	−0.014	−0.016	*0.251*	0.106	−0.062	0.090	*0.369*	3	−0.012	0.844
4. facial emotion identification task	[0.088, 0.303]	[−0.262, −0.044]	[−0.126, 0.098]	–	*0.357*	−0.036	*0.123*	*0.128*	0.062	0.001	4	0.093	0.065
5. facial emotion-rating task	[0.246, 0.442]	[−0.181, 0.042]	[−0.128, 0.096]	[0.255, 0.451]	–	0.020	*0.261*	*0.134*	*0.113*	0.001	5	*0.239*	< 0.001
6. acceptance of stereotyping	[−0.020, 0.201]	[0.132, 0.343]	[0.143, 0.353]	[−0.147, 0.076]	[−0.092, 0.131]	–	0.048	0.009	0.017	*0.144*	6	0.046	0.336
7. category-based trait-rating task	[0.334, 0.517]	[0.102, 0.315]	[−0.006, 0.215]	[0.012, 0.232]	[0.153, 0.362]	[−0.064, 0.159]	–	*0.179*	−0.015	0.103	7	*0.310*	< 0.001
8. cognitive reflection test	[−0.005, 0.216]	[−0.140, 0.084]	[−0.173, 0.050]	[0.017, 0.237]	[0.023, 0.243]	[−0.103, 0.121]	[0.068, 0.285]	–	0.048	**−***0.167*	8	−0.002	0.966
9. need for cognition	[−0.127, 0.096]	[−0.023, 0.199]	[−0.022, 0.200]	[−0.050, 0.172]	[0.001, 0.222]	[−0.094, 0.129]	[−0.127, 0.097]	[−0.065, 0.158]	–	*0.159*	9	−0.045	0.343
10. faith in intuition	[−0.088, 0.136]	[0.262, 0.456]	[0.268, 0.462]	[−0.111, 0.112]	[−0.110, 0.113]	[0.033, 0.252]	[−0.009, 0.212]	[−0.273, −0.056]	[0.048, 0.266]	–	10	−0.058	0.263
	correlation (point estimate with 95% CI)												
11. age	0.005 [−0.106, 0.116]	0.093 [−0.018, 0.202]	*.144* [0.034, 0.251]	0.012 [−0.099, 0.123]	0.022 [−0.089, 0.133]	0.034 [−0.077, 0.145]	*−0.197* [−0.301, −0.088]	0.100 [−0.011, 0.209]	0.085 [−0.026, 0.195]	0.031 [−0.080, 0.142]	11	0.079	0.102
12. sex (male = 1, female = 2)	*0.143* [0.032, 0.250]	0.019 [−0.093, 0.129]	0.016 [−0.095, 0.127]	*0.167* [0.057, 0.273]	*0.187* [0.078, 0.292]	−0.075 [−0.184, 0.037]	0.065 [−0.047, 0.174]	*−0.120* [−0.228, −0.009]	−0.038 [−0.148, 0.073]	*0.141* [0.030, 0.248]	12	0.085	0.081
13. extreme response style measure	*0.351* [0.250, 0.445]	0.086 [−0.025, 0.195]	0.038 [−0.074, 0.148]	0.001 [−0.110, 0.112]	*0.226* [0.118, 0.329]	−0.051 [−0.161, 0.061]	*0.161* [0.051, 0.267]	−0.068 [−0.178, 0.043]	0.054 [−0.058, 0.164]	*0.113* [0.002, 0.221]	13	*0.271*	< 0.001
14. impression management subscale	0.070 [−0.041, 0.180]	0.100 [−0.011, 0.209]	−0.002 [−0.113, 0.109]	−0.008 [−0.119, 0.103]	*0.202* [0.093, 0.307]	−0.085 [−0.195, 0.026]	0.043 [−0.069, 0.153]	−0.018 [−0.129, 0.093]	*0.178* [0.068, 0.283]	0.012 [−0.099, 0.123]	14	−0.050	0.309

^a^Physiognomic Belief Scale [29].

^b^Jaeger's questionnaire of physiognomic belief [53].

#### 3.3.7. Correlational analysis

Table 3 summarizes the partial correlations among the variables of interest, wherein participants' age, sex, extreme response bias and social desirability bias were controlled. The generalized FBTI extremity had small to moderate partial correlations with the scores on the Physiognomic Belief Scale, the two tasks measuring facial emotion recognition ability and the category-based trait-rating task. The partial correlation between the FBTI extremity and the Cognitive Reflection Test score remained weak even when participants were split into those who had (*r* = 0.105, 95% CI [–0.067, 0.272]) and had not (*r* = 0.013, 95% CI [–0.136, 0.162]) taken the test before. We also conducted multiple regression analysis to explain the generalized FBTI extremity with respect to the other variables (not preregistered). Statistically significant standardized partial regression coefficients at the 5% level were obtained for the scores on the Physiognomic Belief Scale, facial emotion-rating task, category-based trait inference task and Extreme Response Style Measure. Thus, the generalized FBTI extremity was positively associated with physiognomic belief, facial emotion recognition ability and stereotype endorsement that were not attributable to its positive relationship with extreme response bias. Zero-order correlation analysis also revealed a tendency for female participants to score higher than male participants regarding generalized FBTI extremity. This sex difference may be mediated by women's advantage in facial expression recognition [57], since its effect size approached zero in multiple regression analysis, wherein explanatory variables included facial expression recognition ability.

## 4. General discussion

### 4.1. Summary of main findings

Studies 1 and 2 consistently showed that those who make extreme face-based judgements on a certain trait also tend to make extreme judgements on other traits, providing evidence for generalized FBTI extremity. The results are in contrast with the evolutionary perspectives on FBTI [22–24] that its inter-individual variation would be trait-specific. Instead, they are more compatible with psychology theory-based predictions that there are several variables that could enhance the FBTI extremity across traits. In partial accordance with our hypotheses, generalized FBTI extremity was positively associated with physiognomic belief, facial emotion recognition ability and stereotype endorsement, but not with cognitive miserliness. In short, we demonstrate that there are individuals who have a temporally stable disposition to draw extreme conclusions about various traits of others from facial appearance as well as their psychological characteristics. These findings and continued research into this issue will contribute to a better understanding of the nature of the excessive impact of face on social decision making, sometimes called *face-ism* [6], and to an identification of a prime target population for its intervention [15,16].

### 4.2. Generalized face-based trait inference extremity

Our analyses ensured that the generalized FBTI extremity reflects true individual differences in social cognition and is not merely a by-product of methodological artefacts. First, the well-established trustworthiness-by-dominance model of facial impressions [17,25,26] was reproduced from the present data, dismissing the possibility that our task format prompted participants to judge faces solely on a single dimension. Second, high cross-trait correlations of the FBTI extremity were demonstrated even after controlling for non-specific response biases such as extreme response style [51] and socially desirable responding [52]. Third, we showed that the generalized FBTI extremity had good test–retest reliability over a 2-month interval. Electronic supplementary material, text S2 also includes a complementary study we conducted to determine whether the temporal stability of the generalized FBTI extremity was explained by response biases. While this study exhibited a somewhat lower test–retest correlation of generalized FBTI extremity (*r* = 0.699, 95% CI [0.586, 0.786]) than Study 1, the value changed only slightly after controlling for extreme response style and socially desirable responding (*r* = 0.681, 95% CI [0.563, 0.772]). These results indicate that the generalized FBTI extremity is a temporally stable disposition.

Concerns may be raised if our attempts to rule out a response-bias account are sufficient. First, the extreme response bias when rating others' faces may not be well captured by the bias when rating self-descriptive verbal statements (i.e. Extreme Response Style Measure [51]). To address this issue, extreme response bias was quantified using data from the face-based trait-rating task. Taking trustworthiness as an example, 10 faces that were not classified as either stereotypically trustworthy- or untrustworthy-looking (i.e. whose mean trustworthiness rating ranked between 11 and 20 out of 30 faces) were selected separately from male and female faces. Then, for each participant, the ratings were recoded such that higher values reflected a greater tendency to choose extreme categories on the scale (i.e. ratings of 6, 5, 4, 3, 2 and 1 were recoded into 3, 2, 1, 1, 2 and 3, respectively), and the recoded ratings were averaged across the selected 20 faces. Bias scores for other trait judgements were defined in the same way, and the averages of trait-specific scores were treated as an overall index of extreme response bias in the face-based trait-rating task. Even when controlling for this additional measure of extreme responses, high cross-trait correlations of the FBTI extremity were reproduced. Moreover, while the association of generalized FBTI extremity with physiognomic belief was not significant, its associations with facial emotion recognition ability and stereotype endorsement were replicated. Second, it may be argued that, since the face-based trait-rating task had participants rate all traits at once for each face, lazy response behaviours, such as frequently choosing the same values on the rating scale, and/or strategic response behaviours, such as trying to provide a consistent (plausible) pattern of ratings, could have inflated cross-trait correlations of FBTI extremity. Regarding this point, we would like to note that in the face-based trait-rating task, desirable traits appeared almost evenly on the left and right sides (figure 1*a*)—in other words, there were reverse items for each face, and the order of the trait dimensions was randomized across trials. Therefore, it is unlikely that a lazy response leads to high cross-trait correlations. In addition, this counterbalanced and randomized setting would require significant effort when viewing the scale to provide a consistent pattern of ratings. Thus, we used survey completion times as viable measures of participant effort [58] and examined their correlations with the variables of interest in the present research. We found that the generalized FBTI extremity did not correlate positively with completion times, whereas it negatively correlated with squared completion times. Albeit a post hoc speculation, it is possible that participants finishing the survey at a moderate pace made the strongest effort, which might have elevated cross-trait correlations of FBTI extremity. This interpretation could explain the negative correlation between the squared completion times and generalized FBTI extremity. However, controlling for completion times (including their squares) did not change our main findings. Based on these additional analyses (see electronic supplementary material, text S3 for details), we believe that the generalized FBTI extremity would not be an artefact of the response bias.

It is also worthwhile to discuss the distinctions between explicit rating tasks akin to the one used in this study and those tasks measuring FBTI in a more indirect fashion. A seminal work proposing the trustworthiness-by-dominance model of facial impressions was based on the data of explicit trait ratings of faces [17]. Subsequent influential studies that replicated and extended the two-dimensional model ([23,25,34]; for a review, see [26]) and recent pursuits of perceiver effects on FBTI [3–5,21,59] also used explicit rating tasks. Therefore, our results indicating the presence of individuals who explicitly form extreme facial impressions across traits would be a valuable addition to the literature on FBTI, which relies heavily on data from explicit rating tasks. On the other hand, facial impressions have also been assessed by more indirect measures, such as the latency and dynamics of responses related to trait inference [60–62] and decision making in simulated social settings [53,63,64]. In general, explicit and indirect measures assess related but distinct constructs [65], and one measure is not necessarily superior to the other. For example, perceived trustworthiness of a given face is often measured using trust games in which participants are asked to decide how much money they are willing to entrust to the owner of the face [53,63,64]. Trust games may appear to provide a better indicator of trustworthiness impressions than explicit rating tasks given that money has an objective unit of measurement (e.g. British Pound), whereas the meaning of verbal anchors on a rating scale (e.g. *very trustworthy*) is subjective and may vary across participants. However, the relationship between monetary decisions in trust games and trustworthiness impressions of game partners is actually not straightforward and depends on participants, because those decisions are influenced by various factors, such as the participant's general trust, preference for fairness and betrayal aversion [66]. In addition, the use of indirect measures is usually recommended when explicit measures are likely to be biased by people's tendency to hide socially undesired responses [65]. Importantly, such social desirability bias was found to have a nearly null correlation with generalized FBTI extremity in the present study. Therefore, we suggest that the use of an explicit rating task does not significantly undermine our findings. Moreover, a fruitful direction for further research is to investigate individual differences in FBTI using indirect measures, since even their basic characteristics (e.g. how large and temporally stable the individual differences are) remain unknown.

### 4.3. Correlates of generalized face-based trait inference extremity

The positive association between the physiognomic belief and the generalized FBTI extremity means that those who believe that various traits can be inferred from faces tend toward extreme FBTI, which is in line with previous work [29]. This association was more clearly shown with the Physiognomic Belief Scale [29] than with the other questionnaire [53], perhaps because the former contains more items than the latter (14 versus 3) and, consequently, has higher scale reliability (Cronbach's *α* = 0.918 versus 0.817), and because the items of the former directly refer to the trait dimensions in the face-based trait-rating task, while those of the latter do not. In any case, the correlations were small, ranging from 0.104 to 0.171. The weak correlations may be reasonable considering that the questionnaires we used assessed the strength of the physiognomic belief without referring to any stereotypical facial cues to trait inference. It would thus be interesting in future research to measure the strength of explicit verbalizable beliefs about commonly held face–trait relations (e.g. ‘A kind-hearted person has big, round eyes’ [9]) and examine its links with the FBTI extremity.

It is noteworthy that we successfully demonstrated in an adult population that those who were adept at recognizing facial expressions tended toward extreme FBTI. While such a relationship is consistent with an emotion overgeneralization account of FBTI [17,22,30,31], a recent developmental study showed that emotion comprehension skills (including facial emotion recognition ability) covaried with the extremity of face-based trustworthiness inference only in 5-year-old children and not in 7-year olds [20]. The authors opined that covariation was absent in older children because their emotion comprehension skills were more developed and thus less variable. In the present study, the facial emotion-rating task scores were more variable than the facial emotion identification task scores, which approached a ceiling effect, and the former scores had a larger correlation with generalized FBTI extremity than the latter. These results indicate that if individual differences in facial emotion recognition are quantified using sensitive tests [35,36], their association with FBTI can be detected even in adulthood.

Our research partially supports the tendency toward extreme FBTI among people who endorse stereotypes. Specifically, the category-based trait-rating task scores moderately and positively correlated with generalized FBTI extremity, while the Acceptance of Stereotyping Questionnaire [39] scores hardly did. In addition, the two measures, which were assumed to reflect the same construct (i.e. stereotype endorsement), were almost entirely uncorrelated with each other. A critical difference between the two indices is that whereas the rating task explicitly deals with stereotypes about social categories that are closely connected with FBTI (i.e. smiling people [17,33], babies [37] and women [38]), the questionnaire does not specify any social categories. Thus, if participants who self-identified as White (i.e. the majority of the study participants) answered the questionnaire with stereotypes about racial outgroups in mind [67], it is natural that the answers showed little correlation with the FBTI extremity, considering the exclusive use of Caucasian faces (i.e. ingroup faces) in the face-based trait-rating task.

The fact that the hypothesized positive correlations between generalized FBTI extremity and cognitive miserliness were not observed may be taken as evidence of the cognitive impenetrability of FBTI [68]. However, a caveat is that the face is the only information available to perform the face-based trait-rating task. This could obscure possible facilitative effects of cognitive miserliness on FBTI, since even those participants who doubted the validity of their intuitive impression of the face—our measures of cognitive miserliness are supposed to assess such a tendency (not) to rely on intuition—had no choice but to make a face-based inference. Thus, it is important to examine whether cognitive miserliness boosts reliance on facial appearance in trait inference when more diagnostic but cognitively taxing cues (e.g. a record of previous cooperative and cheating behaviours [69]) are available.

### 4.4. Limitations

This study has some other limitations worthy of mention. First, a direct and more extensive investigation into the causal mechanisms of generalized FBTI extremity is important in future research. Our major contribution is the demonstration of high cross-trait correlations of the FBTI extremity, or the existence of a generalized FBTI extremity, providing new directions for and constraints on further studies and theorizing about FBTI. As an initial attempt to explain the generalized FBTI extremity, we proposed and examined several variables that can potentially foster it. However, those variables were a small subset of possible causal factors, and the present study provided only partial and correlational evidence for our hypotheses. Second, different researchers have adopted different methods to score FBTI performance [3,20,21,29,59]. For example, when agreement scores [21] (see the Introduction for a definition) were computed using the present data, they had high correlations (above 0.7) with extremity scores. However, the scatter plots between the two types of scores showed that there were larger variations in extremity scores when the agreement scores were higher (see electronic supplementary material, text S4 for details). In other words, individuals with higher agreement scores can have both low and high extremity scores. Thus, FBTI extremity is not only related to agreement scores but also contains distinct perceiver characteristics. Future investigations need to explore a set of quantifications that can efficiently and comprehensively capture individual differences in FBTI. Third, our surveys started with a block of socio-demographic questions, since collecting certain socio-demographic data (i.e. current residence, primary language and age) was necessary to confirm participants' eligibility to participate in the surveys. These questions might have directed participants' attention to their age, sex and ethnicity, which in turn could have influenced how they approached the subsequent social cognitive tasks. However, the participants' age and sex were controlled for in the statistical analyses presented here. In addition, the results remained virtually unchanged when we analysed the data of only White participants (i.e. a large majority of participants). Therefore, we suggest that even if participants' age, sex and ethnicity had been activated by socio-demographic questions, they should have exerted little impact on our findings. Finally, our studies only targeted native English speakers in the USA, given the availability of well-normed materials in English, leaving cross-cultural similarities and differences unexplored [25].

## Data Availability

Preregistration forms, study materials, datasets and analysis codes can be accessed via the following link: https://osf.io/axtvz. Supplementary material is provided in the electronic supplementary material [70].
